# The Reliever Reliance Test: evaluating a new tool to address SABA over-reliance

**DOI:** 10.1038/s41533-024-00389-4

**Published:** 2024-11-05

**Authors:** Zoe Moon, Alan Kaplan, Vincent Mak, Luis Nannini, Tonya Winders, Amy Hai Yan Chan, Holly Foot, Rob Horne

**Affiliations:** 1 Spoonful of Sugar Ltd, a UCL Business Company, London, UK; 2 Family Physician Airways Group of Canada, Clinical Lecturer University of Toronto, Toronto, ON Canada; 3 Imperial College NHS Healthcare Trust, London, UK; 4 E Peron Hospital, Universidad Nacional de Rosario, Santa Fe, Argentina; 5 Global Asthma and Allergy Patient Platform, Vienna, Austria; 6 School of Pharmacy, University of Auckland, Auckland, New Zealand; 7 School of Pharmacy, University of Queensland, Brisbane City, QLD Australia; 8 Centre for Behavioural Medicine, University College London, London, UK

**Keywords:** Patient education, Asthma

## Abstract

Over-use of SABA is associated with poor asthma control and greater risk of exacerbations and death. Identifying and addressing the beliefs driving SABA over-reliance is key to reducing over-use. This study aimed to assess the utility, impact and acceptability of the Reliever Reliance Test (RRT), a brief patient self-test behaviour-change tool to identify and address SABA over-reliance. Patients with asthma who completed the RRT in Argentina were invited to an online survey exploring the acceptability of the RRT, and its impact on patients’ perceptions of SABA and intention to discuss asthma treatment with a doctor. 93 patients completed the questionnaire. The RRT classified 76/93 (82%) as medium-to-high risk of SABA over-reliance (a mindset where SABA is perceived as the most important aspect of asthma treatment), with 73% of these reporting SABA overuse (3 or more times a week). 75% intended to follow the RRT recommendations to review their asthma treatment with their doctor. The RRT is acceptable to patients and was effective at raising awareness of, identifying and addressing SABA over-reliance and encouraging patients to review their treatment with their doctor.

## Introduction

Short-acting beta2-agonists (SABA) have been the main symptomatic treatment for people with asthma for over 40 years, usually prescribed alongside an inhaled corticosteroid (ICS) preventer inhaler^1^. However, whilst SABA may provide effective short-term symptom relief, it has no anti-inflammatory properties and therefore does not treat the underlying cause of asthma. Evidence has shown that frequent SABA use is associated with increased risk of exacerbations and asthma related deaths^2–5^. In April 2019, the Global Initiative for Asthma (GINA) updated treatment guidelines to recommend that asthma symptoms should not be treated with SABA monotherapy and reiterated the need for greater emphasis on anti-inflammatory agents, even when managing symptoms^6^.

Both patients and clinicians have had many years of experience with SABA as the recommended reliever treatment for asthma. This may represent a “custom and practice”^7,8^ where use of 3 or more SABA cannisters per year, which is now perceived as over-use^7^, may not have historically been seen as problematic. The SABA Use in Asthma (SABINA) program collected data from over a million individuals and found approximately 40% of patients across all asthma severities were prescribed or received 3 or more SABA inhalers per year, defined as SABA over-use^7^. Understandably, many patients have developed emotional attachments to their SABA and believe it to be the best way to manage their asthma, due to the quick symptom relief they experience and the prioritisation of symptom relief over prevention of future attacks^8–11^. Patients may also be unaware that frequent SABA use indicates poor asthma control^9^. This state of mind has been referred to as SABA over-reliance, and represents strong perceived personal need for SABA, anxiety about not having access to it, and preference for SABA over other treatments^8^. SABA over-use also typically occurs in parallel with the underuse of ICS^12^, potentially due to negative ICS attitudes, including strong steroid related concerns, and the perception that ICS treatment is only needed during exacerbations^8,13^. These perceptions about both ICS and SABA affect patient behaviour and therefore need to be elicited and addressed for behaviour to change in line with established asthma guidelines.

The SABA Reliance Questionnaire (SRQ) is a valid and reliable measure of patients’ perceptions of their personal need for SABA to manage their asthma^8^. High scores indicate a perception that SABA is the most important aspect of asthma treatment and a high reliance on SABA to manage asthma. The Reliever Reliance Test (RRT) is a self-completed behaviour change tool that incorporates the SRQ and adds personalised behaviour change messaging to inform patients about the dangers of SABA overuse, encourage them to reflect on their perceptions of SABA, and alert them to fact that relying on SABA to manage their asthma may not represent the best treatment for them. It encourages them to discuss their asthma treatment with a healthcare professional (HCP), if applicable^14^. The RRT incorporates the SRQ in a form that is self-completed by the patient. The patient’s SRQ score is then fed back to the patient, along with information about SABA over-reliance that is designed in a way that is likely to challenge attitudes to SABA and discourage over-reliance on SABA^14^.

It also provides advice about the correct use of SABA inhalers. Patients can also download the results of the test to take to their doctor, nurse, or pharmacist to support a conversation about their asthma management. We anticipate patients at low risk of SABA over-reliance may also want to discuss their treatment to ensure it is best for them.

The RRT was developed in collaboration with patients, clinicians, and the International Primary Care Respiratory Group (IPCRG) and is currently being used in over 20 different countries. It is available in digital and print format and can be accessed via online sites or through healthcare professionals.

The RRT has the potential to improve clinical practice by helping to address misplaced beliefs that put patients at risk of SABA over-reliance. This can prepare patients for conversations with healthcare professionals, giving clinicians a better opportunity to discuss treatment options with patients in time limited consultations. A previous study has shown that the messaging within the RRT changes beliefs about SABA^14^, but little is known about whether this leads patients to make any changes to their treatments or if it prompts them to seek help.

## Aim

The aims of this study were to

(1) assess its utility as a screening tool to identify patients at risk of SABA over-reliance

(2) conduct a preliminary evaluation of efficacy in terms of:

(A) changing patients’ attitudes towards SABA and their asthma and asthma treatment more generally

(B) motivating patients to consult with their doctor regarding their asthma treatment

(3) assess the acceptability of the RRT delivered through a web-based channel

## Results

**Fig. 1:**
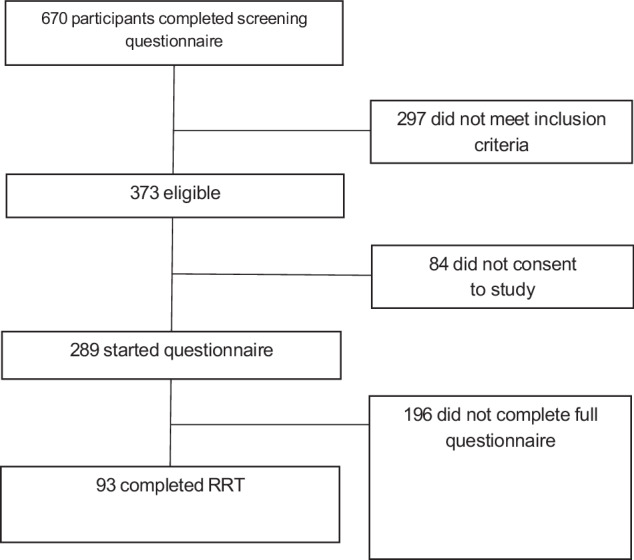
Participant flow diagram.

670 participants completed the online screening questionnaire. 297 did not meet the inclusion criteria (e.g. no reported diagnosis of asthma/no SABA prescription) and 84 did not give informed consent. 289 participants were eligible and consented, and of these 109 (38%) completed some of the questionnaire, and 93 completed the RRT (32%) (Fig. 1).

Table 1 shows participant characteristics. Just under half of participants were female (49%), and the majority were aged between 18–25 (30%) or 26–40 (30%). Most participants reported having asthma for over ten years (66%). The majority were prescribed ICS alongside their SABA (63%), with 34 (37%) on SABA monotherapy.

**Table 1:** Participant characteristics.

	*N* (%)
Gender	
Female	44 (49%)
Male	46 (51%)
Age	
18–25	27 (30%)
26–40	27 (30%)
41–55	20 (22%)
56–70	15 (17%)
Over 71	1 (1%)
Time since asthma diagnosis	
<1 year	4 (4%)
1–5 years	12 (13%)
5–10 years	10 (11%)
Over 10 years	61 (66%)
Can’t remember	6 (7%)
Other prescribed asthma medications	
No other treatments (SABA monotherapy)	34 (38%)
SABA + ICS	58 (62%)
GINA symptom control assessment^a^	
Uncontrolled	61 (67%)
Partly controlled	26 (29%)
Well controlled	4 (4%)

Ns do not add to 109 due to missing data.

*ICS* Inhaled Corticosteroids, *LABA* long-acting beta-agonist.

^a^Global Initiative for Asthma Global Strategy for Asthma Management and Prevention (2019) Available from: https://ginasthma.org/wp-content/uploads/2019/06/GINA-2019-main-report-June-2019-wms.pdf.

Most participants gave answers suggesting uncontrolled (67%) or partly controlled (29%) asthma, with only 4% having well controlled asthma on the GINA Symptom Control Assessment.

SABA over-use: 48 (52%) participants reported using their SABA inhaler 3 or more times a week, with 33 (36%) using it over 5 times a week.

**Fig. 2:**
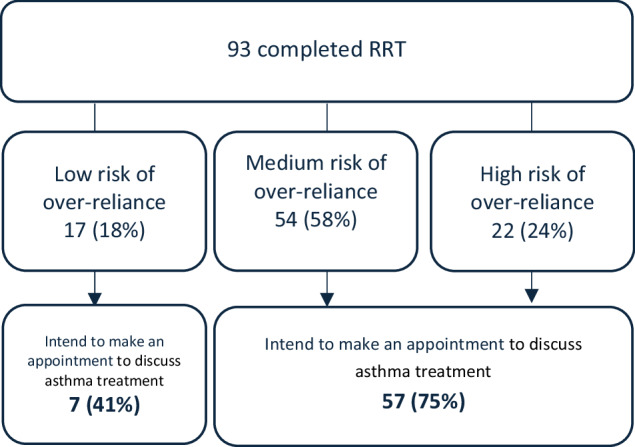
Categorisation of patients and intentions to visit doctor.

SABA over-reliance: The RRT classified 17 (18%) participants at low risk of SABA over-reliance, 54 (58%) at medium risk and 22 (24%) at high risk of over-reliance (Fig. 2).

Relationship between over-use and over-reliance: Of those who were at medium or high risk of over-reliance (*n* = 76), 73% reported using their SABA inhaler 3 or more times a week over the past month. Of those who were using their SABA inhaler 3 or more times a week (*n* = 67), 89% also had an attitude of over-reliance.

Participants were asked how important their SABA inhaler was to them on a scale of 0–10. The mean score was 8.34 (SD = 1.97), with 42 participants (45%) scoring 10 out of 10. Participants were asked how worried they were about using their SABA inhaler on a scale of 0–10. Mean scores were 6.34 (SD = 3.07). 54% agreed that using their SABA inhaler less than 3 times a week would be difficult for them. 39% agreed that using their SABA inhaler less than 3 times a week would be reasonable.

RRT scores were significantly related to asthma control (χ^2^ (2) = 8.75, *p* < .013). None of the patients who had well controlled asthma (*n* = 4) were at high risk of SABA over-reliance. 19% of those with partly controlled asthma (*n* = 26), and 28% of those with poorly controlled asthma (*n* = 61) were at high risk of SABA over-reliance.

**Fig. 3:**
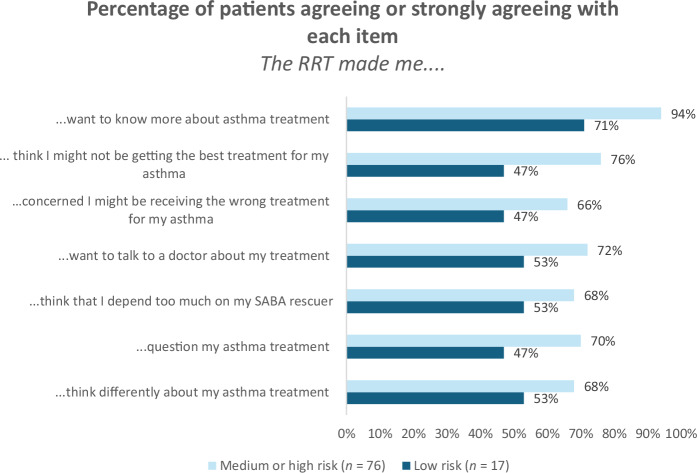
Change in patient attitudes after completing the RRT. Note: RRT Reliever Reliance Test; SABA Short acting beta2 agonists.

Participants were asked the extent to which the RRT had made them feel differently about their asthma treatment. High rates of agreement were seen across all items for those at medium-to-high risk (66–94%). 68% agreed that the RRT made them think they depend too much on their SABA inhaler. 72% agreed the RRT made them want to talk to a doctor, and 76% agreed the RRT made them think they might not be getting the best treatment for their asthma (Fig. 3). Agreement of these items was higher in those who were classed as medium or high risk of over-reliance compared to those who were classed as low risk of over-reliance.

**Fig. 4:**
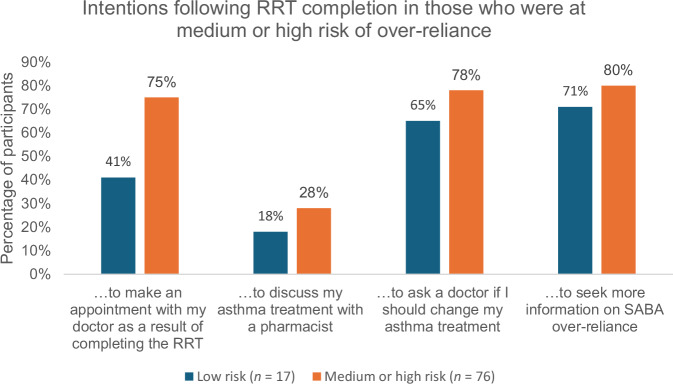
Percentage of participants intending to take action following the RRT.

The RRT was effective at encouraging participants to take action. Of those who were at medium or high risk of over-reliance, and who were therefore recommended to see a doctor, 75% (*n* = 57) said they intended to make an appointment with a doctor as a result of completing the RRT (Fig. 4). 28% (*n* = 21) intended to discuss their treatment with a pharmacist, 78% (*n* = 59) intended to ask a doctor if they should change their asthma treatment, and 80% (*n* = 61) intended to seek more information on SABA over-reliance. 41% (*n* = 7) of those who were classed as low risk of over-reliance also intended to make an appointment with their doctor as a result of completing the RRT.

When looking specifically at those whose asthma was poorly controlled (*n* = 61), 75% (*n* = 46) intended to make an appointment to discuss their asthma treatment with a doctor.

**Fig. 5:**
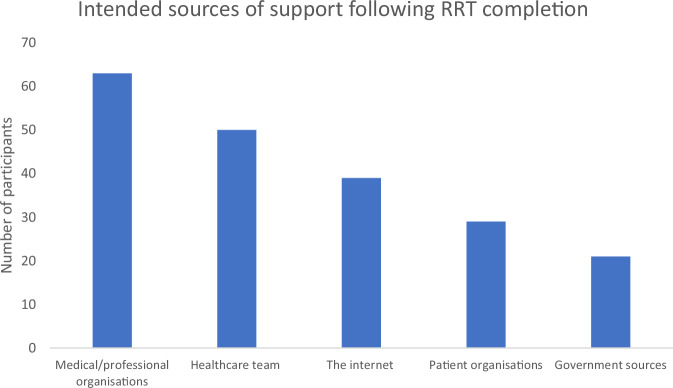
Intended sources of support following RRT completion.

Participants were asked where they would seek support following the RRT completion (Fig. 5). 63 (68%) indicated they would seek support from medical/professional organisations, 50 (54%) from their healthcare team, 39 (42%) from the internet, 29 (31%) from patient organisations, 21 (23%) from government sources.

75% of participants agreed that the RRT was helpful, and 72% agreed that it was important to them. 25% (*n* = 23) felt that the RRT didn’t affect them. Those that said the RRT did not affect them were less likely to be over-using their SABA. 30% (*n* = 7) used their SABA three or more time a week, compared to 52% in the full sample. Only 10% of participants felt that the RRT did not make sense to them.

34 participants (37%) reported being prescribed SABA monotherapy, which may have resulted in particularly high perceptions of SABA reliance (as it is the only treatment available to them personally). We questioned whether the relatively high rates of SABA over-reliance within the sample could have been mainly driven by patients on SABA monotherapy. To address this, we conducted a sensitivity analysis in which we compared scores within the SABA monotherapy sample and those that were prescribed SABA in addition to ICS (*n* = 58; Supplementary Table 1). Asthma control was poor in both groups, but worse in the SABA monotherapy sample, with 71% having poorly compared asthma compared to 65% of the ICS and SABA group. The risk of over-reliance was higher in the SABA monotherapy group, with 94% at medium to high risk of over-reliance compared to 74% in the ICS and SABA group and 82% across the full sample.

## Discussion

This is the first study to show that completion of the self-test RRT tool encourages patients to review their asthma treatment with their doctor. It demonstrates the positive impact of brief tailored messages on attitudes towards asthma treatment and how this can address SABA over-reliance. Given that reducing SABA use is a key element of asthma treatment guidelines^6^, the RRT could help to ensure patients receive optimal guideline driven treatment. Our results show that the tool is acceptable to patients (72–75% found it helpful and important) and that it encourages patients to think differently about their SABA. This may better prepare them for a consultation in which changes to their asthma treatment could be made.

Around two thirds of the patients in this study have poorly controlled asthma and report being very attached to their SABA, even in patients who are also prescribed ICS. Over half have been living with asthma for over ten years. This is a group of patients who may be thought of as resistant to change and may be particularly hard to convince^11^. However, we have shown that the RRT can motivate even these patients to seek help. The results show very high rates of intention to take action, with three quarters of patients indicating that they intend to make an appointment to discuss their treatment with a healthcare professional. It is likely that not all patients will follow through on this intention, and it was not possible to measure patient behaviour in this study. Nonetheless, these are very high rates of intention, especially compared with similar behaviour change interventions^15,16^.

Interestingly, even within those who are told they are at low risk of over-reliance, around half appear to have had their perceptions about asthma changed and 41% intend to discuss their treatment with their doctor. This may be due to the very brief messaging provided to low-risk patients, which informs them that SABA only treats the symptoms of asthma and not the underlying cause. This messaging, independent of information about their personal risk of SABA over-reliance, has prompted people to want to review their asthma treatment with their doctor. Future work should explore the impact on low-risk patients and ensure the test does not cause anxiety.

The SABA monotherapy cohort are interesting. It may seem entirely obvious that patients who report only being prescribed one treatment for asthma are likely to be overly reliant on this treatment. However, it is important to recognise that the SABA Reliance Questionnaire (SRQ) component of the RRT differentiates between people based on their perceived necessity for SABA, with very high necessity beliefs being indicative of over-reliance (e.g., *SABA is the best way to control my asthma*). The clinical relevance of this is that such patients may be reluctant to consider alternative approaches to managing their asthma, e.g. the addition of ICS maintenance treatment and/or an ICS-LABA as anti-inflammatory reliever therapy (AIR) or maintenance and reliver therapy (MART).

Changes made by the RRT to the patient’s mindset around asthma and its treatment pave the way HCPs to be able to discuss alternative treatment options with patients. As it currently stands, HCPs may be reluctant to discuss these issues with patients, due to time constraints or lack of confidence in identifying or addressing excessive SABA use themselves^17^. The RRT may therefore be useful in clinical practice as a tool to help to identify those at risk of over-reliance, and to prepare patients for conversations around the risks associated with over-using SABA and the importance of alternative treatments such as anti-inflammatory treatment. This increases patient receptiveness to messaging, reduces the burden on clinicians, and provides them with an opportunity to have a meaningful discussion about treatment options.

There are several limitations with this study. Firstly, there was a risk of selection bias, as participants had to agree to participate, and a relatively large percentage of patients dropped out before completing the survey. Participants who dropped out may have been at lower risk of SABA over-reliance. Patients also had to have a level of digital literacy in order to take part in the study. However, the included participants represent an important group of patients who are over-reliant on their SABA and have poorly controlled asthma, which is the target population group for the RRT. Secondly, the sample size was small. Future studies should test this effect in a larger trial. Third, some patients may be relying on their SABA due to issues around accessibility or cost, and the RRT does not explore these factors. Some patients were prescribed SABA monotherapy, and their lack of a preventer therapy will likely contribute towards their over-use or over-reliance on their SABA. From our sensitivity analysis we conclude that patients on SABA monotherapy are more reliant on SABA than those who are also on ICS treatments. However, the differences were not substantial and high rates of over-reliance were still seen in the ICS sample and so the results still highlight a need for the RRT. Fourth, we relied on self-reported diagnosis of asthma rather than clinical diagnosis. Finally, the study only assessed changes to perceptions and intentions. We do not know how many of these patients followed up on their intention and visited their doctor. Future research is needed to explore the impact of the RRT on behaviour change, to test in larger samples and explore the effectiveness of the RRT across multiple countries.

Nonetheless, although limited in scope, this pilot study suggests that the RRT holds promise as a low-cost intervention to change patients attitudes to SABA, educate them about asthma treatment and encourage patients to seek treatment reviews with HCPs. Results indicated the RRT was well accepted by the patients who completed it. It is likely that the tool could be easily rolled out and widely applied, as it can be completed very quickly and can be delivered through advertisements and social media, or given the tool directly by their HCP^18^. This could include the pharmacy team who are the last HCPs patients interact with before taking their treatment home. When delivered directly with a HCP, it is a brief, pragmatic way of identifying the beliefs that drive SABA over-reliance and provides HCPs with specific targets to discuss in consultations. If the patient completes the RRT outside of the consultation, it serves as a tool to alert the patient to the risks of over-using SABA and help them come to terms with the fact that there may be a better way of treating their asthma.

Future research should explore the most effective strategy to support HCPs, so they can continue on from the work of the RRT and appropriately counsel patients towards meaningful behaviour change. The results from this study show that even patients who score at low risk of over-reliance on the RRT intend to visit their doctor to discuss their asthma treatment, as they have been informed that there are better treatment options available. Therefore, the HCP and healthcare system needs to be ready to help these patients and to provide suitable support. Data should also be collected to assess whether campaigns increase demand in such a way that HCPs do not have the resources to deal with. Furthermore, special care will have to be taken to train HCPs in countries where alternatives to SABA are not readily available or too costly. The RRT has currently only been tested in a few countries. Further cross-cultural adaptation and validation is needed.

The RRT is effective at identifying and addressing SABA over-reliance and motivating people to seek help, in a sample of patients who are largely overly reliant on their SABA and have poor asthma control. This study supports its potential usefulness as a tool in clinical practice. Motivating patients to reduce their SABA usage should help more patients achieve optimal outcomes. The current study only measured intentions to seek help. More research is needed to explore the extent to which the RRT will change help-seeking behaviour and medication usage.

## Methods

**Fig. 6:**
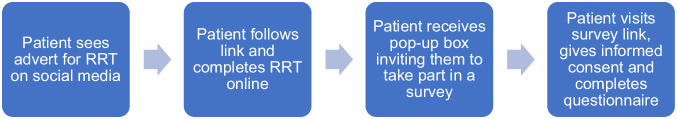
Participant flow across study.

This was a cross-sectional survey of patients with asthma in Argentina, who were invited to participate after they had voluntarily completed the RRT online (asmazero.com). Patients were alerted to the RRT through a social media campaign spearheaded by a pharmaceutical company. After completing the RRT, participants were invited to take part in a short questionnaire to share their experiences with and attitudes towards the RRT. The questionnaire was hosted on an external website (https://www.qualtrics.com) and took approximately 15 min to complete. See Fig. 6 for an overview of the patient recruitment process.

As this is a service evaluation, ethical approval was not required. However, all research was conducted in accordance with the British Healthcare Business Intelligence Association (BHBIA) legal and ethical guidelines for healthcare market research, and The Code of Ethics of the World Medical Association (Declaration of Helsinki).

Adults aged over 18 who identified as having asthma and being prescribed a SABA were eligible to participate in the survey. All eligible participants were shown an information sheet and informed consent was taken from all participants using an online form. The following outcome measures were taken:

Patient demographics: The following clinical and demographic information was self-reported: time since asthma diagnosis, prescribed treatments for asthma, age and gender.

Questions within RRT tool:

1. SABA Over-reliance: Participants were asked to complete the SABA Reliance Questionnaire (SRQ), which forms the first part of the RRT. The SRQ is a 5-item validated scale to assess over-reliance to SABA^8^. Items are scored on a five-point Likert scale from Strongly Agree to Strongly Disagree. The scores from these five items are totalled and participants are scored as being at low risk (<10) medium risk (11–17) or high risk (18–25) of over-reliance. The Spanish translation of the SRQ shows good psychometric properties^19^.
2. SABA usage: Participants are also asked how often they have used their SABA inhaler over the last four weeks (*none, once or twice a week, 3 times a week, between 4-5 times a week, more than 5 times a week*). Using SABA three or more times a week was defined as over-use.

Participants also answered the following questionnaires as part of the evaluation:

GINA symptom control tool^20^: Patients were asked in the past 4 weeks if they have had daytime asthma symptoms more than twice a week, any waking in the night due to asthma, any activity limitations due to asthma and if they needed to use their reliever more than twice a week. Patients who ticked none of these were classed as well controlled, those who ticked 1-2 were classed as partly controlled and those who selected 3 or 4 of these were classed as uncontrolled.

Beliefs about SABA: Participants were asked two questions to assess their beliefs about SABA: *How important is your SABA inhaler to you* and, *How worried are you about using your SABA inhaler*. Both items were based on the validated and widely used Beliefs about Medicines Questionnaire (BMQ^21^) and were scored on a scale from 0 to 10. Participants were also asked how easy or difficult it would be for them to use their SABA fewer than 3 times a week.

Impact of the RRT on attitudes towards asthma and asthma treatment: Participants were asked to rate the extent to which they agreed to 8 items around the impact that the RRT had on their thoughts about asthma treatment (e.g., *The RRT made me feel differently about the way I use my asthma treatment, The RRT makes me question which asthma treatment is best for me*). Data was collected on a 5-point Likert scale (strongly agree to strongly disagree).

Intentions following the RRT: Participants were asked to indicate if they intended to take any of the following actions after completing the RRT: *Make an appointment to discuss my asthma treatment with a doctor, discuss my asthma treatment with a pharmacist, ask a doctor if I should change my asthma treatment, seek more information on SABA over-reliance, do nothing*. Participants could select multiple options.

Acceptability of the RRT: Participants were asked to rate the extent they agreed that the RRT: *was helpful, was important, had no effect on me at all, did not make sense to me*. Data was collected on a 5-point Likert scale (strongly agree to strongly disagree).

Data were cleaned and analysed in IBM SPSS Statistics. Descriptive analyses (means, SD or *n* %) were carried out for all variables (median, IQR used for non-normally distributed variables). A risk of reliance score was calculated for each patient based on their scores on the SRQ (see methods section for scoring). The percentages of participants intending to take each action following RRT completion (e.g. visiting their doctor) were calculated. The percentage agreeing or strongly agreeing with items around changes to asthma treatment perceptions were calculated. The relationship between asthma control and risk of reliance was tested using Chi Squared.

### Reporting summary

Further information on research design is available in the Nature Research Reporting Summary linked to this article.

## Supplementary information

Reporting Summary

Supplementary Table 1 01980R1

## Data Availability

The datasets used and/or analysed during the current study are available from the corresponding author on reasonable request.
