# Supplementary material for: The Reliever Reliance Test: evaluating a new tool to address SABA over-reliance
Source: NPJ Prim Care Respir Med. 2024 Nov 5;34:36. doi: 10.1038/s41533-024-00389-4 (PMC11538409; doi:10.1038/s41533-024-00389-4)
Supplement: Supplementary file 2 — Supplementary Table 1 01980R1 [file 41533_2024_389_MOESM2_ESM.docx]

**Supplementary Table 1**

Comparison of patients prescribed SABA monotherapy (n=35) vs those prescribed ICS and SABA (n=58)

|  | Full sample  (n=93) | SABA monotherapy (n=35) | ICS and SABA (n=58) | Chi Squared test |
| --- | --- | --- | --- | --- |
| Asthma control  Well controlled  Partly controlled  Poorly controlled | 4 (4%)  26 (29%)  61 (67%) | 0 (0%)  10 (29%)  24 (71%) | 4 (7%)  16 (28%)  3 (65%) | *p*=.286 |
| SABA over-use  3 or more times a week  More than 5 times a week | 48 (52%)  33 (36%) | 18 (53%)  14 (41%) | 37 (66%)  19 (33%) | *p*=.594 |
| SABA over-reliance  Low risk  Medium risk  High risk | 17 (18%)  54 (58%)  22 (24%) | 2 (6%)  21 (60%)  12 (34%) | 15 (26%)  33 (57%)  10 (17%) | *p=.023* |
| Intentions following RRT (in medium to high risk)  Make an appointment with a doctor  Ask a doctor if they should change treatment  Seek more information | 57 (75%)  59 (78%)  61 (80%) | 23 (72%)  25 (78%)  24 (75%) | 34 (79%)  34 (79%)  37 (86%) | *p*=.968  *p*=.745  *p*=.198 |
| RRT acceptability  Helpful  Important  Did not effect them  Did not make sense to them | 70 (75%)  67 (72%)  23 (25%)  9 (10%) | 25 (74%)  25 (74%)  5 (15%)  3 (9%) | 43 (76%)  42 (72%)  18 (31%)  6 (10%) | *p*=.872  *p*=.952  *p*=.114  *p*=.789 |
